# The Effect of Surface Electroplating on Fragment Deformation Behavior When Subjected to Contact Blasts

**DOI:** 10.3390/ma16155464

**Published:** 2023-08-04

**Authors:** Yuanpei Meng, Yuan He, Chuanting Wang, Yue Ma, Lei Guo, Junjie Jiao, Yong He

**Affiliations:** School of Mechanical Engineering, Nanjing University of Science and Technology, Nanjing 210094, Chinactwang@njust.edu.cn (C.W.);

**Keywords:** contact blast, coatings, fragment deformation behavior, dynamic response, protection performance

## Abstract

Preformed fragments can deform or even fracture when subjected to contact blasts, which might lead to a reduction of the terminal effect. Therefore, to solve this problem, the effect of surface electroplating on the fragment deformation behavior under contact blasts was analyzed. Firstly, blast recovery tests were carried out on uncoated and coated fragments. After the contact blast, the two samples produced different deformation behaviors: the uncoated fragments were fractured, while the coated fragments maintained integrity. The tests were simulated by finite element simulation, and the deformation behavior of the different samples matched well with the test results, which can explain the protective effect of the coating after quantification. In order to further reveal the dynamic behavior involved, detonation wave theory and shock wave transmission theory in solids were used to calculate the pressure amplitude variation at the far-exploding surface of the fragments. The theoretical results showed that the pressure amplitude of the uncoated samples instantly dropped to zero after the shock wave passed through the far-exploding surface, which resulted in the formation of a tensile zone. But the pressure amplitude of the coated samples increased, transforming the tensile zone into the compression zone, thereby preventing the fracture of the fragment near the far-exploding surface, which was consistent with the test and simulated results. The test results, finite element simulations, and theories show that the coating can change the deformation behavior of the fragment and prevent the fracture phenomenon of the fragment. It also prevents the material from missing and a molten state of the fragment in the radial direction by microscopic observation and weight statistics.

## 1. Introduction

The fragments had different degrees of deformation and even fracturing behavior after blast loading, which would affect the integrity [1,2], the terminal effect [3], and the initial velocity [4,5] of the preformed fragments. However, preformed fragments were often considered rigid when their dispersion characteristics and terminal effect were studied under different modes of detonation (concave [6], unsymmetrical [7], and conventional [8]). This has led to poor agreement between many studies and test results.

The deformation and fragmentation behavior of preformed fragments under contact blasts actually belonged to the problem of the dynamic response of the material under a high strain rate [1]. Many scholars have studied the fracture behavior of different metallic materials under high strain rates. The spalling phenomenon of common metals has been studied by scholars in the last century [9]. The fracture characteristics of metals at high strain rates could also be predicted by using simulation software [10,11]. In addition to the macroscopic study and prediction of fracture behavior, the microstructure changes of materials during high strain rate loading have also been studied [12,13]. Electron microscopic analysis of the recovered preformed fragments revealed that the aggregation of microporosity caused the failure [1,2]. The fragments fractured when the tensile stress generated inside the fragment was greater than the fracture stress [14].

Therefore, two methods have been used to prevent the deformation, fracture, and even fragmentation behavior of preformed fragments under the blast: adding linings and improving the mechanical properties of the fragments. To ensure the completeness of the fragments during the blast loading, the lining was provided between the fragments and the explosive to attenuate the shock wave and the load of the primary explosive [3,15]. The presence of the lining could increase the pulse width and reduce the deformation speed of the shock wave, but this would affect the initial velocity and terminal effect [16]. For example, the mechanical properties of metallic materials could be enhanced by adding different particles [17,18]. The dynamic failure of Fiber Reinforced Metal Tubes (FRMTs) under inner blast load was experimentally investigated [19]. The mechanical properties of metals were also improved by microstructure optimization [20]. These methods on the dynamic mechanical properties of the material could avoid fracture failure under a high strain rate. But these studies were hardly universal and needed to be more cost-effective. 

Protective coatings, such as polyurea [21] and polymers [22], were also suggested to reduce the deformation and fracture of metals under blast. However, this approach was still influenced by the fracture strength of the metal material itself due to the low impedance of the polyurea and polymers coatings themselves, which cannot change the direction of the shock wave at the back-blast surface [23,24]. The material would still produce deformation and even fracture at a sufficiently high strain rate. Therefore, there is an urgent need to investigate a generalized method that can change the deformation behavior and prevent it from fracturing at high strain rates without affecting its initial velocity and terminal effect.

This study was initiated to protect the fragments under contact blasts via an alternative approach: surface electroplating of high-impedance coatings. The effect of surface electroplating and impedance matching on fragment deformation behavior subjected to contact blasts was thus investigated.

## 2. Experimental and Simulation Methods

### 2.1. Preparation Methods

**Figure 1:**
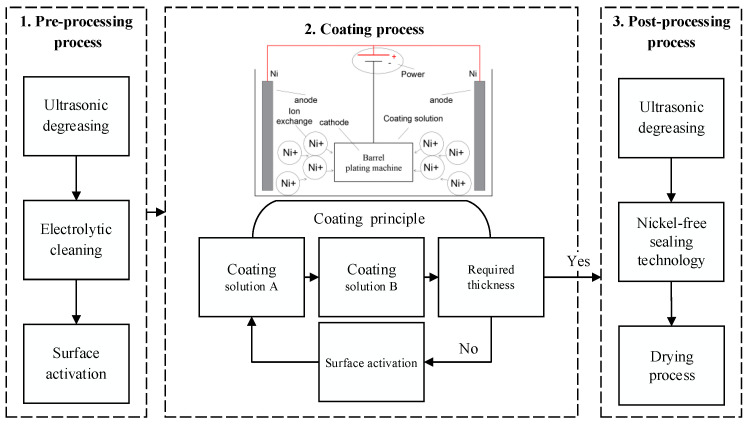
Flow chart of coating preparation procedure.

The uncoated test samples (UC-10L) were made with commercial purity zirconium in the shape of a cylinder (*φ*11 mm × *h*13 mm). The coated sample (C-10L) was prepared by coating a thick layer of nickel (commercial purity) on the uncoated sample. Since the matrix in the coated sample is the uncoated sample, it is referred to as a zirconium fragment when comparing the discussion of the matrix (in C-10L) and the uncoated sample (in UC-10L). The procedure of coating preparation is shown in Figure 1. Nickel metal with a thickness of ~1 mm was coated outside the uncoated test samples: Firstly, the surface of the preformed fragments was degreased and activated. Then, the preformed fragments were coated with coating solutions A and B (Nanjing WANQING chemical Glass ware & Instrument Co., Ltd. Nanjing, China), in turn, repeated ~40 times until the thickness of the coating reached ~1 mm. The formulations of coating solutions A and B are shown in Table 1, with PH values of 3.4 and 4.5, respectively. The microstructure was observed using the JSM-IT500HR (Tokyo, Japan) scanning electron microscope with a working voltage of 20 KV. 

**Figure 2:**
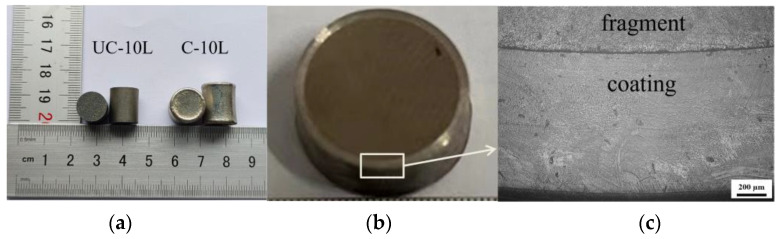
Characterization of samples (UC-10L and C-10L). (**a**) uncoated and coated samples, (**b**) cross-section of the C-10L, (**c**) electron microscope images.

Figure 2a shows the samples before and after coating (UC-10L and C-10L). Figure 2b shows the cross-section microstructure of the C-10L. The interface between the zirconium fragment and the Ni coating is clearly visible, and the two parts are marked in Figure 2c. The hardness of the coating was tested by HV-1000A microhardness tester. The hardness of the coating is 3960 ± 60 MPa, while the hardness of the zirconium fragment was 1270 ± 40 MPa.

### 2.2. Experimental Method

**Figure 3:**
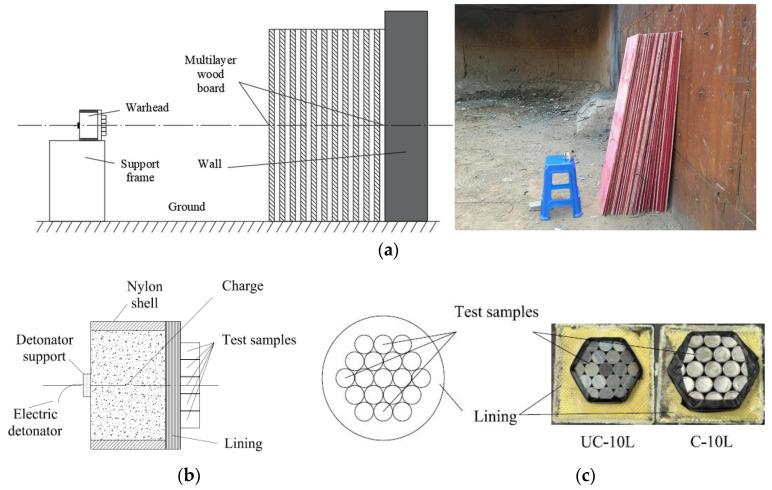
Schematic and layout diagram of the blast recovery test. (**a**) Experimental design, (**b**) Warhead assembly drawing, (**c**) The arrangement of the tests.

Uncoated samples (UC-10L) and coated samples (C-10L) were used in contact explosion tests. A previous study found that the Kevlar/epoxy lining material was beneficial in reducing the fragment deformation degree, preserving the initial velocity of fragments, and maintaining the fragments’ quality integrity [15,25]. The schematic diagram and physical diagram of the test layout are shown in Figure 3, in which the warhead axis is parallel to the horizontal plane, and the multilayer wood boards are placed on the right to recover the test samples after the explosive drive. An appropriate distance was selected to ensure that more fragments were recovered, which was set to 40 cm. The warhead structure is shown in Figure 3b. The simulated warhead (*φ*72 mm × *h*50 mm) was a condensed charge (8701). The tests were carried out using single-point detonation in the center of the end face. The placement of fragments is shown in Figure 3c. It was sealed with adhesive tape to ensure the tight arrangement of fragments. Because of the weak strength of the tape, the binding force on the fragments scattering during the contact explosion drive could be ignored. The specific method was as follows: A column booster (*φ*15 mm × *h*5 mm) was built in the center of the left end of the explosive, and an electric detonator was connected to the column booster. The charge, lining (Kevlar/epoxy composite), and the two test samples fit snugly with each other.

The lining was made as follows: First, Kevlar was cut into a square with a side length of 72 mm and put into the box one by one. Afterward, the resin glue was applied to each layer of Kevlar, and a heavy object was used to press it to ensure a tight fit between Kevlar layers [25,26]. The test arrangement is listed in Table 2. The recovered fragments were then microscopically observed by the FEI Quanta 250F.

### 2.3. Simulation Model

**Figure 4:**
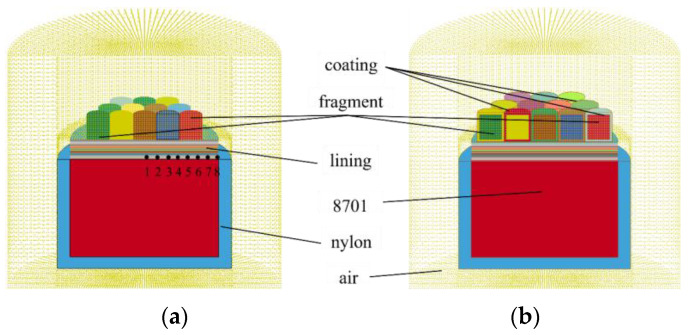
Simulation modeling of the blast recovery tests. (**a**) UC-10L, (**b**) C-10L.

The simplified warhead simulation model is shown in Figure 4, which consists of the charge, lining, shell, and test samples. Charge (*φ*72 mm× *h*50 mm) used 8701, and lining (*φ*72 mm× *h*10 mm) was above the charge. The shell used nylon, wrapped in the cylindrical surface of the explosive and the bottom; its thickness was 2 mm. This paper used the ALE algorithm to numerically simulate the blast driving process by finite element simulation(FEM). The calculation process ignored the impact of the column booster on the detonation. Charge, detonation products, air, lining, and samples were used in the multi-matter Euler grid, and Kevlar/epoxy composite material was selected as the lining. To analyze the change of wave pressure at different locations of Kevlar/epoxy composite media, eight reference points are set equidistantly at the intersection of the lining and charge, as shown in Figure 4. The material parameters used in the simulation are shown in Table 3, Table 4, Table 5, Table 6, Table 7 and Table 8.

The macroscopic homogeneous model was used to model the Kevlar/epoxy composite in this paper [27], and 054/055 material in the finite element material model was used to reproduce the macroscopic orthotropic anisotropic mechanical properties, including failure criteria for the fiber and the epoxy matrix [28,29]. 

As shown in Figure 4, the lining is modeled with a single-layer mesh of 40 layers, and the number of meshes per layer in the axial direction is 1. The adjacent Kevlar layers needed to apply bonding forces due to the presence of the epoxy medium. Similarly, in the plated samples, there is a bond between nickel and zirconium, so the binding contact element was introduced in the finite (*CONTACT_AUTOMATIC_SURFACE_TO_SURFACE_TIEBREAK). This keyword allowed the Kevlar of two adjacent layers to remain bound at the beginning of the FEA [29,30]. But the binding keyword automatically degraded to face-to-face contact when the detonation wave pressure reached the destructive forces of tension and compression defined above. 

## 3. Experimental and Simulation Results

### 3.1. Axial Deformation and Fracture

**Figure 5:**
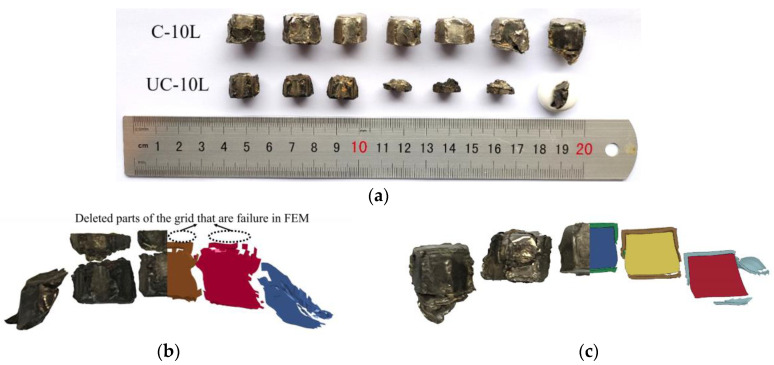
Comparison of recovered samples and simulations. (**a**) Typical samples recovered by the two methods, (**b**) Comparison of simulation and test results of UC-10L, (**c**) Comparison of simulation and test results of C-10.

After the contact blast, the UC-10L and C-10L samples were recovered, as shown in Figure 5a. The UC-10L samples fractured and were divided into two main pieces (long and short) in axial length. Their fracture surfaces were not flat, and the average value was taken when measuring the length, while the recovered C-10L samples were complete, and no fractures were found. Comparison of simulation and test results on the morphology of fragments are shown in Figure 5b,c. After the fracture failure of the UC-10L samples occurred, the fracture location was selected as the benchmark for comparing the two since the mesh in the simulation would be deleted where the blank position in the simulation is deleted by the FEM after the mesh failure. The consistency between the test results and the simulation of both samples was acceptable, indicating that the simulation can predict the results of the test to some extent. 

**Figure 6:**
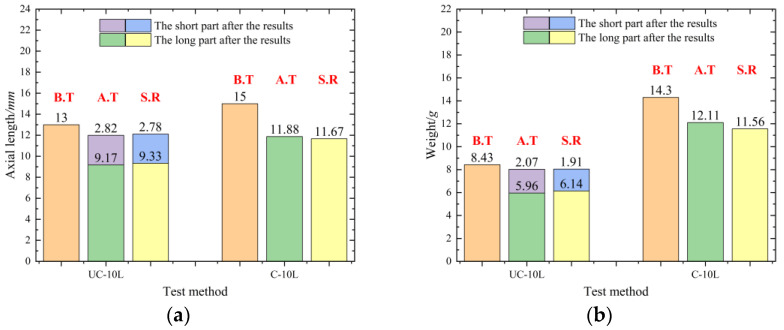
Axial length and weight of samples after blast. (**a**) Axial length of samples after blast, (**b**) Weight of samples after blast. (“B.T” means “before the test”, ”A.T” means “after the test”, and “S.R” means “simulation results”).

The axial lengths of recovered typical samples are shown in Figure 6a, and the height of the sample is lower than its original height (13 mm for the sample before the UC-10L samples and 15 mm for the sample before the C-10L samples). For the UC-10L samples, the complete samples were composed of long and short samples [5]. The sum of the average value of long samples (9.17 mm) and the average value of short samples (2.82 mm) was 11.99 mm. The sum was lower than that of the original sample (13 mm).

The average axial length of the samples recovered from the test method of C-10L was 11.88 mm. The value was not only lower than 15 mm (before the C-10L samples) but even lower than the sum of the axial length of the fracture samples and the short samples after the C-10L samples. The compression ratio of the C-10L samples was around 20.8%, higher than the compression rate received by the UC-10L samples (around 7.8%). Combining the comparison results in Figure 5, it was speculated that the coating changed the deformation behavior. 

The weights of recovered samples are shown in Figure 6b. The sum of the average value of long samples (5.96 g) and the average value of short samples (2.07 g) was 8.03 g. The sum was less than 8.43 ± 0.05 g (before the test), similar to the statistical results of axial length. This was because the fragment also occurred in the radial direction. The specific analysis will be discussed in Section 3.2.

In summary, after comparing the axial length and weight of the two sets of experiments, it could be preliminarily inferred that due to the presence of the coating, the deformation behavior of the fragment under the contact explosion was changed. 

### 3.2. Radial Local Fragmentation and Melting

**Figure 7:**
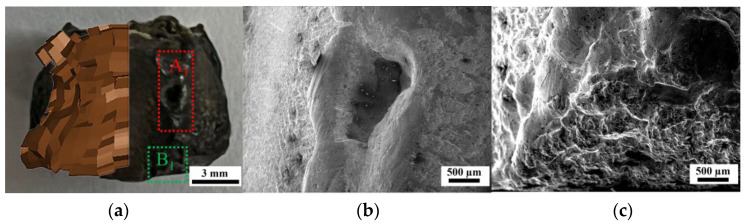
Optical and electron micrograph of the sample recovered from the test method of UC-10L. (**a**) Comparison of simulation and test results, (**b**) Electron micrograph of position A_1_, (**c**) Electron micrograph of position B_1_.

**Figure 8:**
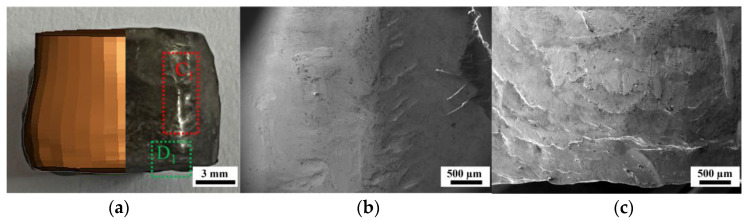
Optical and electron micrograph of the sample recovered from the test method of C-10L. (**a**) Comparison of simulation and test results, (**b**) Electron micrograph of position C_1_, (**c**) Electron micrograph of position D_1_.

In order to compare the protective effect of the coating on the zirconium fragment radially, the surface nickel coating was manually removed from the recovered samples of C-10L. The radial local fragmentation and melting situation are shown in Figure 7a and Figure 8a, and there are six straight “ridges” in the circumferential axis of all recovered samples, which correspond to the hexagonal shape of their near-explosive surface. This is because although the samples are placed as close to each other as possible, there are still small gaps between the fragments in the radial direction. Therefore, the adjacent fragments in the radial direction collide to form a “ridge” when the shock wave passes through the samples [5].

As shown in Figure 7b, the cylindrical surfaces of the recovered uncoated samples have a lot of material missing and a molten state. This is due to the “welding effect” caused by the collision between adjacent fragments in the test method of UC-10L, so there is a molten state on the “ridge” [5], and due to the fragmentation caused by the detonation action of the fragment, resulting in the separation of the fragments welded together and the material is thus partially missing [1,5]. As shown in Figure 8b, because the samples from the test method of C-10L have the coatings, even if the coatings come off in this condition, the nickel replaces the impact “welding effect” of the fragments in the radial direction. Thus, no molten state and material is missing on the surface in the radial direction, which protects the fragments from fracture in the radial direction. 

As shown in Figure 7c, the surfaces of the bottom of the “ridge” of the samples recovered from the UC-10L samples have traces of upward flow in addition to the molten state [1,25]. But as shown in Figure 8c, the surfaces of the bottom of the “ridge” of the C-10L samples only have a transverse texture produced by compression. The reason is that the surface of the UC-10L samples was not protected by the coating, and the high-temperature gas flow (explosive detonation product) generated by the explosion caused it to be prone to upward plastic flow [25]. The surface of the C-10L samples was coated to replace this plastic flow.

**Figure 9:**
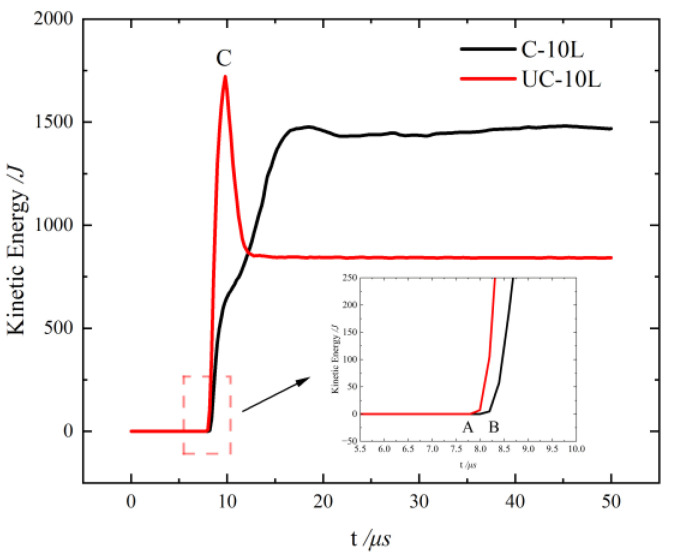
Kinetic energy–time variation curves of the zirconium fragments.

Thus, combined with the weight statistics, it shows that the coating not only causes a change in the deformation pattern of the zirconium fragments in the axial direction but also prevents it from local fragmentation and melting in the radial direction. To further illustrate the protective effect of the coating on the zirconium fragments, Figure 9 shows the kinetic energy–time variation curves of the zirconium fragments in UC-10L and C-10L. As shown in points A and B of Figure 9, the kinetic energy increase in C-10L lags behind that of UC-10L. This is because Figure 9 shows the kinetic energy change curve of zirconium fragments, and the shock wave reaches the coating first in the C-10L. The kinetic energy change curve of zirconium fragments for UC-10L decreases sharply at point C, which is due to the fracture of zirconium at this time. This is due to the fact that the part of the fracture is deleted directly in the simulation, which leads to this situation. This also proves that the presence of the coating ensures the integrity of the zirconium fragments and retains more of their kinetic energy.

## 4. Analysis and Discussion

### 4.1. Detonation Wave Transmitted to the Lining

The impact effect of high-speed detonation products on solids was different from that of general static loads. Thus, it must be studied from a dynamic perspective and wave concept [14]. The experiment in this study was the case of an axial drive fragment, so the radial detonation wave was ignored. The wavefront of the detonation wave was spherical, so the detonation wave was considered oblique incidence in the lining. According to the angle of incidence and the magnitude of the wave impedance, the transmission reflection generated by oblique incidence at the interface was divided into normal oblique incidence, informal oblique incidence, and Prandtl–Meyer (P-M) expansion. 

**Figure 10:**
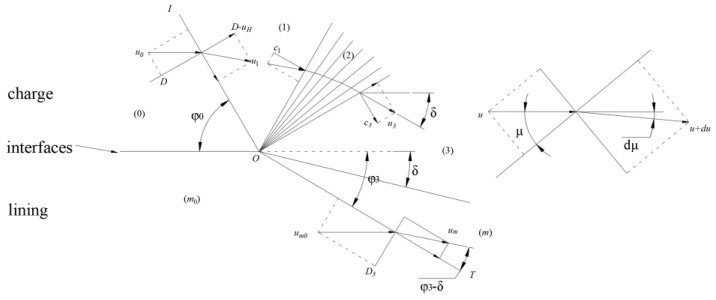
Schematic of the blast wave flowing into the lining.

The wave impedance of Kevlar/epoxy lining is less than that of explosives, so it belongs to P-M expansion at the interface between the explosive and lining. The flowing image is shown in Figure 10, where *OI* is an oblique detonation wave front, the angle between *OI* and the interface of the contact medium is *φ*_0_; *OT* is the oblique transmission shock wave front in the incoming medium, and the angle between *OT* and the initial interface of the medium is *φ*_3_; the lining medium is deformed under the action of detonation, and the angle between the interface after the medium moves and the initial interface of the medium is *δ*. In this way, the oblique detonation wave, oblique reflection expansion wave, oblique transmission shock wave, and interface divide the entire flow into six regions: (0) area is unexploded, (1) area is the area of detonation product after oblique detonation wave, (2) area is the expansion area of detonation product, (3) area is the area of detonation product after expansion, (*m*_0_) region is the initial medium, and (*m*) region is the area of medium disturbance after the oblique transmission shock wave [14,25]. 

This study assumed that the detonation wave was stable and self-sustaining detonation in the explosive. The state parameters of the detonation wave generated are taken from the parameters of the C-J point. The wavefront is a circular arc in the two-dimensional case. The effect of circumferential blast wave transverse reflection on the axial direction is ignored. The relationship between the parameters in the (3) region and the known parameters can be obtained according to the conservation and flow law of the detonation wave front, as shown in the following two equations:

(1) $$M_{3}^{2}=\frac{M_{1}^{2}+\frac{2}{k-1}}{{\left[\frac{(k+1)\rho_{m0}\sin\varphi_{3}}{b\rho_{0}\sin\varphi_{0}}\left(\frac{\sin\varphi_{3}}{\sin\varphi_{0}}-\frac{a}{D}\right)\right]}^{\frac{k-1}{k}}-\frac{2}{k-1}}$$

(2) $$\begin{array}{c} \left[\sqrt{\frac{k+1}{k-1}}arctg\sqrt{\frac{k-1}{k+1}\left(M_{3}^{2}-1\right)arctg\sqrt{M_{3}^{2}-1}}\right]-\\ \left[\sqrt{\frac{k+1}{k-1}}arctg\sqrt{\frac{k-1}{k+1}\left(M_{3}^{2}-1\right)arctg\sqrt{M_{3}^{2}-1}]+\theta }\right]\\ =arctg\left[\frac{\left(1-\frac{asin\varphi_{0}}{D\sin\varphi_{3}}\right)tg\varphi_{3}}{b+\left(b-1+\frac{asin\varphi_{0}}{D\sin\varphi_{3}}\right)tg^{2}\varphi_{3}}\right] \end{array}$$

where *M*_3_ is the Mach number of the (3) zone, and *M*_1_ is the Mach number of the (1) zone; *ρ*_0_ and *ρ_m*0*_* are the initial densities of the explosive and lining, respectively; *k* is the thermal insulation index of the explosive detonation product; *a* and *b* are the empirical constants of impact compression of the lining medium; *D* is the explosive detonation rate; $\varphi_{0}$ is the angle between the incident and the initial interface of the medium; $\varphi_{3}$ is the angle between the transmitted wave and the initial interface of the medium; and *θ* is the flow folding angle.

The calculation for the Mach number *M*_1_ is shown in the following equation:

(3) $$M_{1}=\sqrt{1+{\left(\frac{k+1}{k}\right)}^{2}ctg^{2}\varphi_{0}}$$

The calculation for the flow bending angle *θ* is shown in the following equation:

(4) $$tg\theta =\frac{tg\varphi_{0}}{1+k\left(1+tg^{2}\varphi_{0}\right)}$$

The calculation for the adiabatic index *k* of the explosive detonation product is shown in the following equation:

(5) $$\{\begin{array}{l} k=1.25+k_{0}\left(1-e^{-0.546\rho_{0}}\right)\\ k_{0}=\frac{\overset{i=1}{\underset{L}{\sum }}\frac{\mu_{i}}{M_{i}}}{\overset{i=1}{\underset{L}{\sum }}k_{0i}M_{i}} \end{array}$$

where *k*_0_ is the total adiabatic index of the mixed explosive detonation product; *ρ*_0_ is the charge density (g/cm^3^) of the mixed explosive; *μ* is the mass percentage of component *i* of the explosive mixture; and *M_i_* is the molar mass of component *i* of the explosive mixture.

The composition of the explosives used in the test is shown in Table 9. The adiabatic index (*k*) of 8701 explosives can be calculated as 2.85 [25]. The values of the parameters required for the above calculation are shown in Table 10.

Combining Equations (1)–(5), the parameters *M*_3_ and *φ*_3_ in the (3) region can be obtained.

The (*m*) zone parameter can be obtained from Equations (6)–(8):

(6) $$\frac{\rho_{m0}}{\rho_{m}}=1-\frac{1}{b}\left(1-\frac{asin\varphi_{0}}{D\sin\varphi_{3}}\right)$$

(7) $$p_{m}=\frac{\rho_{m0}D^{2}\sin\varphi_{3}}{b\sin\varphi_{0}}\left(\frac{\sin\varphi_{3}}{\sin\varphi_{0}}-\frac{a}{D}\right)$$

(8) $$u_{m}=\frac{D\sin\varphi_{3}}{\sin\varphi_{0}}{\left[{\left(1-\frac{1}{b}+\frac{asin\varphi_{0}}{bD\sin\varphi_{3}}\right)}^{2}+tg^{2}\varphi_{3}\right]}^{\frac{1}{2}}$$

where *ρ_m_*, *p_m_*_,_ and *u_m_* are the state parameters of the post-wave medium. The transmitted wave velocity is: $D_{m}=\frac{D\sin\varphi_{3}}{\sin\varphi_{0}}$.

**Figure 11:**
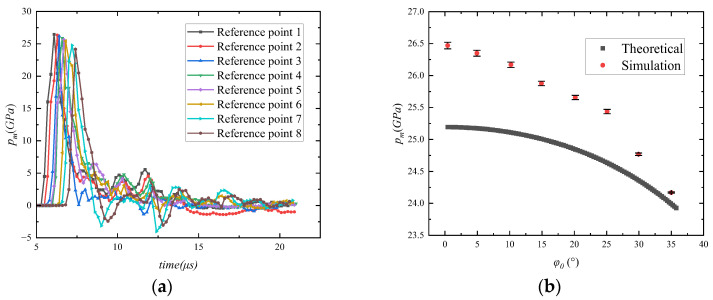
Pressure amplitude changes in the lining. (**a**) Pressure variation curve of reference points, (**b**) Comparison of theoretical and simulated pressure value.

Eight reference points were chosen based on the angle between the detonation wave and the lining. As shown in Figure 11a, the variation of pressure amplitude for eight references on the lining plane, the time to reach the pressure peak increases as the angle between the shock wave and the axis becomes larger. The pressure peaks at these eight reference points are compared with the theoretically calculated curves, as shown in Figure 11b. Finite element simulation using macroscopic Kevlar/epoxy lining modeling method with an error of 6% or less. The anastomosis is good and can reflect the pressure change in the lining to a certain extent. 

Therefore, the macroscopic model established in this paper can be employed to calculate the pressure value at various positions of the lining. This pressure value could be regarded as the input pressure transmitted to the samples.

### 4.2. Detonation Wave Transmitted to the Samples

When the shock wave travels through the liner and reaches the samples, the shock wave is assumed to be a plane wave when they propagate, which is based on the following: 1. The uncoated fragments and the coated fragments are in contact with the lining. 2. The explosive core is along the axis of the samples. 3. A one-dimensional wave is used to simplify the analysis when near the symmetry axis of complex space. 4. As shown in Figure 11a,b, the detonation wave at different angles is introduced into the lining with little effect at different locations.

Thus, the one-dimensional plane strain wave correlation theory is applied to analyze the propagation of shock waves in a multilayer medium to analyze the deformation behavior of fragments in two different test samples.

**Figure 12:**
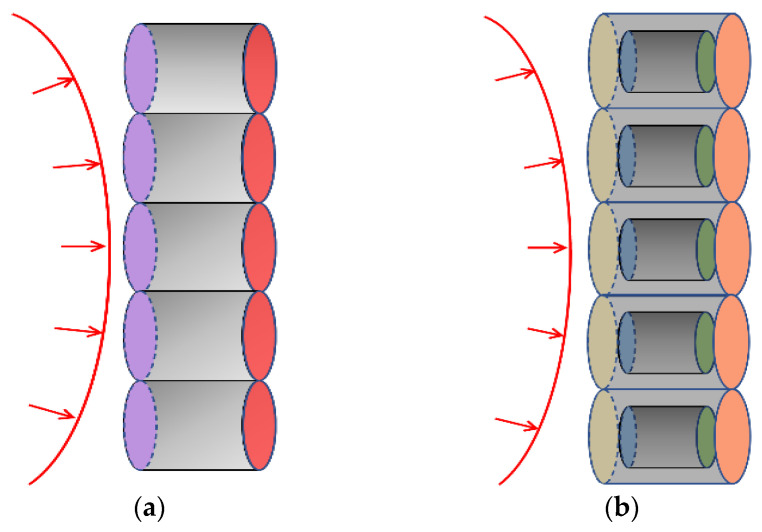
Schematic diagram of different interfaces of the test samples. (**a**) UC-10L, (**b**) C-10L.

Figure 12 is a schematic diagram of the different interfaces of the UC-10L and C-10L samples. The arrows in Figure 12 indicate the propagation of the shock wave. Figure 12a is the UC-10L samples, where the purple interface is the interface between the lining and the zirconium fragment, and the red interface is the interface between the zirconium fragment and air. Figure 12b shows the C-10L samples, where the yellow interface is the interface between the lining and the nickel coating, the blue interface is the interface between the nickel coating and the zirconium fragment, the green interface is the interface between the zirconium fragment and nickel coating, and the orange interface is the interface between nickel coating and air. 

As the impact impedance of the right medium of purple (yellow) is greater than the impact impedance of the lining on its left side, when the shock wave propagates to the interface of purple (yellow) in Figure 12, the pressure amplitude of the right medium of the purple (yellow) interface will be higher than the initial medium pressure amplitude of the lining.

Then, the propagation theory of shock waves between the two mediums is used to solve the following questions [33]. For the left wave *D_L_* in the lining, taking the left wave as the observation point, the following relationship can be obtained from the fundamental equation of shock wave:

(9) $$u_{a0}-u_{a1}=\sqrt{\left(p_{a1}-p_{a0}\right)\left(v_{a0}-v_{a1}\right)}$$

(10) $$D_{L}+u_{a0}=v_{a0}\sqrt{\frac{p_{a1}-p_{a0}}{v_{a0}-v_{a1}}}$$

Similarly, for the right wave *D_R_* in the right medium, the following relationship can be obtained from the fundamental equation of shock wave:

(11) $$u_{b1}-u_{b0}=\sqrt{\left(p_{b1}-p_{b0}\right)\left(v_{b0}-v_{b1}\right)}$$

(12) $$D_{R}-u_{b0}=v_{b0}\sqrt{\frac{p_{b1}-p_{b0}}{v_{b0}-v_{b1}}}$$

At the interface of the two mediums, it can be obtained by the continuity condition:

(13) $$u_{a1}=u_{b1}\;p_{a1}=p_{b1}$$

In the above equations, subscripts *a* and *b* represent the medium material on the left and right sides of the purple (yellow) interface, respectively, and subscripts 0 and 1 represent the parameters before and after the wave, respectively. The initial parameters (*u_a_*_0_, *ρ_a_*_0_, *P_a_*_0_) of the dielectric material on the left side of the purple (yellow) interface are calculated by the previous Equations (1)–(8). The initial velocity (*u_b_*_0_) in the right medium of purple (yellow) is 0, and the initial density (*ρ_b_*_0_) is a known parameter. The following relationship can be obtained from the Hugoniot relationship between the shock wave velocity and the post-wave particle velocity in the condensed medium:

(14) $$D_{L}=a_{1}+b_{1}u_{a1}\;D_{R}=a_{2}+b_{2}u_{b1}$$

where *a*_1_ and *b*_l_ are the Hugoniot parameters of the left dielectric material of the purple (yellow) interface, and *a*_2_ and *b*_2_ are the Hugoniot parameters of the right dielectric material of the purple (yellow) interface. 

The unknown parameters of the shock wave and particle in the two mediums can be obtained by solving Equations (6)–(14), where *p_b_*_l_ is the initial pressure amplitude obtained by the material under the action of the shock wave. Because this study considered the influence of trans-reflection on the particle parameters of the medium when the shock wave propagated in a different medium, the attenuation of the shock wave in the condensed medium was ignored. 

The solution of the parameters on both sides of the blue interface is similar to the solution on both sides of the purple (yellow) interface. When the shock wave propagates further to the blue interface, the right medium pressure amplitude of the blue interface is lower than the initial medium pressure amplitude on its left side. This is because the shock impedance of the medium on the left side of the blue interface is greater than that on the right side. 

According to the above analysis methods, it is shown that the shock propagation expressions in the shock wave are consistent with the above expressions. The values of the parameters represented by *a*_1_ and *b*_1_ need to be changed to the parameters of the medium on the left side of the blue interface, and *a*_2_ and *b*_2_ need to be changed to the parameters of the material on the right side of the blue interface. Similarly, this method is still used in the green interface. The parameters [14,34] of the different mediums used in the equations are shown in Table 11.

Thus, combined with the results obtained in Section 4.1 (Figure 11), the pressure amplitude in the zirconium fragments can be calculated based on the above equations and parameters.

### 4.3. Changes of Deformation Behavior Caused by the Ni Coating

The air is considered an incompressible medium, and the red interface (UC-10L in Figure 12a) is regarded as a free surface [33]. Thus, the particle state should be solved by the interaction between the shock wave and the free surface. When the shock wave propagating along the medium reaches the free surface, the pressure of the wavefront immediately drops to zero. Then, the medium begins to expand and move forward, and a tensile wave is reflected in the medium compressed by the shock wave. Then, the medium obtains another velocity increment in the original direction of motion. At this time, the particle velocity is doubled; that is, the shock wave is twice the speed criterion of the free surface [35]. Currently, the velocity of the left side of the red (orange) interface is twice as large, and the mass pressure amplitude is zero.

**Figure 13:**
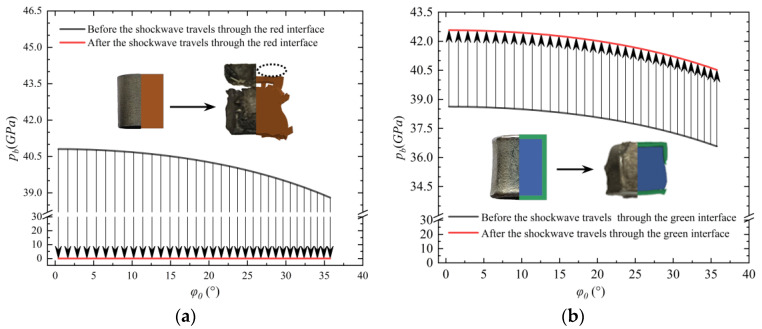
Change of pressure parameters at the different sample surfaces. (**a**) Change of pressure parameters at the red interface, (**b**) Change of pressure parameters at the green interface.

According to the above theoretical analysis, the state parameters of the left and right medium can be obtained when the shock wave travels through several interfaces. Figure 13a shows the variation curves of pressure amplitude with the incident angle before and after passing through the purple interface in the uncoated sample. Figure 13b shows the variation curve of pressure amplitude with incident angle before and after passing through the green interface in the coated sample. The shaded parts in both figures are the variation values of pressure amplitude. The shaded parts in both figures are the value change of pressure amplitude.

Before the shock wave inside the fragments reaches the green interface (coated samples) and red interface (uncoated samples), the pressure amplitude of the mass inside the fragments is greater than 0, as shown in the curves in Figure 13a,b. Because the sample as a whole is subjected to the compression effect generated by the shock wave, its length will be smaller than the length before the detonation in a one-dimensional plane perspective. The length of the samples recovered from the test is shown in Figure 6. It is shown that for all the tests and simulations, the fragment length is less than its original length, which can prove the correctness of the theory.

For the UC-10L samples, when the shock wave is transmitted to the red interface, the pressure amplitude instantly drops to 0. The shaded part of the arrow direction of Figure 13a indicates that it is subjected to stretching when the tensile effect is stronger than its dynamic elastic limit. It produced the fracture phenomenon, so two samples with different fracture lengths were recovered in the UC-10L samples.

For the C-10L samples, when the shock wave to the green interface, as shown in Figure 13b, the pressure amplitude of the mass point on the left side of the interface instantaneously increases. This results that the far-exploding surface will be subject to greater compression than before and the UC-10L samples. The upward arrow in the shaded part of Figure 13b indicates the compression effect on the green interface relative to the rest of the fragments. Therefore, the compression rate of the recovered sample in the C-10L samples (20.8%) was higher than that in the UC-10L samples (7.8%), which proved the consistency between the theoretical analysis and the C-10L samples.

It is reasonable that the deformation behavior will not change when the wave impedance of the coating is lower than that of the fragments but will only reduce the amount of deformation [21,23]. Then, the far-exploding surface of the fragments will have a stretching effect. But the effect of the shock wave unloading caused by stretching is much lower than that of the fragment in the free surface. The effect of stretching gradually increases as the impedance of the coating decreases, and the stretch area decreases.

Therefore, impedance is the key factor that leads to the change in the deformation behavior combined with the above discussion. When there is a layer of medium with an impedance greater than that of the fragment outside the surface of the fragment, it can change the tensile deformation into compressive deformation.

### 4.4. Fracture Mechanism and Calculation of the Fracture Position

The previous section discussed qualitatively the mechanism of fracture of UC-10L samples in terms of the compression and tension zones generated by the shock wave on the far-exploding surface of the fragments. But in fact, the shock wave that causes the force direction to change is a triangular pulse (pressure–time curve) with a wavelength of *λ*. It takes some time for the shock wave to affect the various parameters of the mass inside the fragment, and the time is related to wavelength. Thus, there is a process during the mass pressure amplitude to become 0. This section explains the fracture mechanism from the wavelength perspective and calculates the fracture position.

**Figure 14:**
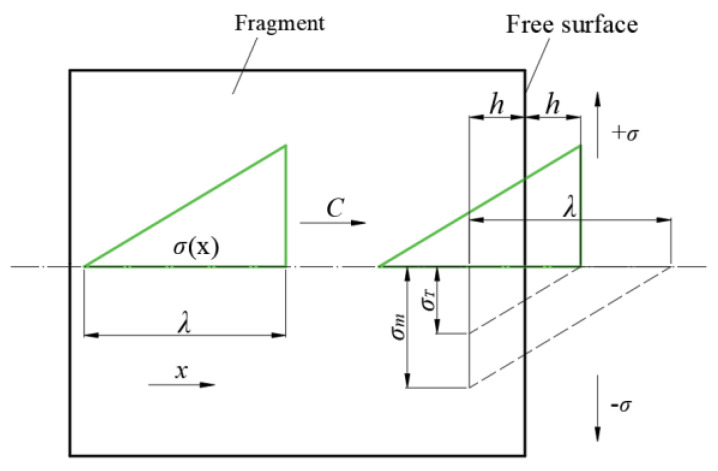
Schematic diagram of the fracture process of the C-10L.

The process principle is shown in Figure 14. When the detonation wave acts on the near-exploding surface, a triangular stress wave will propagate in the fragment, keeping it in a compressed state. According to the principle of a one-way strain plane wave, as the stress wave propagates to the right, the amplitude increases and the wavelength decreases, but the wavefront is still triangular. When the wavefront surface of the stress wave reaches the free surface, a stretching wave comparable to the incident compressional wave will be reflected. The direction of this stretching wave is opposite to the direction of the incident wave. At this time, the incident wave interferes with the reflected wave, and the pressure amplitude gradually drops to 0. Since the tail of the incident wave is still within the free surface, the material within the free surface is kept in compression. After that, the incident wave continues to move outward while the reflected stretch wave moves continuously into the fragment, and the two waves constantly interfere with each other. The material is transferred from the original compression state to the tensile state within the free surface, where the reflected wavefront goes. And as the distance of the tensile wave to the free surface increases, the tensile stress also gradually increases. Fracture begins when the value of tensile stress reaches the critical fracturing stress of the material.

So, in order to facilitate the calculation of the location of the fracture, it is assumed that the shock wave of the fragment is an elastic wave, the wave speed of the fragment of material does not change with the compression, and the wavelength of the compression wave does not change with the distance. That is, the wave speed and wavelength of the shock wave in the fragment are invariant. Therefore, the triangular compressional wave within the fragment propagates without attenuation at the elastic longitudinal wave speed [36]. The peak stress is *σ_m_*, and the dynamic failure stress is *σ_T_*. The incident wave interferes with the reflected wave after the triangular incident stress wave reaches the free surface. Suppose fracture occurs at a distance *h* (mm) from the far-exploding surface, and the geometric relationship can obtain according to Figure 14:

(15) $$\frac{\sigma_{T}}{\sigma_{m}}=\frac{2h}{\lambda }\Rightarrow h=\frac{\sigma_{T}}{2\sigma_{m}}\lambda$$

where *λ* is the wavelength of the compression wave, and *h* is the length of the fracture.

The value of *λ* in the formula is difficult to be found directly, and the empirical fitting is mainly carried out through experiments at this stage [36]. Thus, the approximation method is used in this paper. When the detonation wave travels along the charge to the contact surface, the pressure (*P_m_*) on the fragmentation surface suddenly increases to the maximum value and then drops rapidly. Time elapsed for the pressure amplitude to drop to zero is expressed as the diameter of explosives(*d*) divided by the shock wave velocity (*D_k_*). Thus, the equation for the wavelength (*λ*) is obtained:

(16) $$\lambda =\frac{d}{D_{k}}C$$

where *C* is the longitudinal wave velocity of the shock wave in the material.

(17) $$C_{}=\sqrt{\frac{\frac{E}{3(1-2\nu )}+\frac{2E}{3(1+\nu )}}{\rho }}$$

where *v* is the Poisson’s ratio, *E* is the modulus of elasticity, and *ρ* is the density.

The fracture will occur when the tensile action on pure zirconium under high pressure exceeds the fracture strength. Therefore, the dynamic fracture stress (*σ_T_*) in the equation is used as the fracture strength of the material, and fracture occurs when the fracture strength at this strain rate range is exceeded. However, most of the current methods to obtain the material *σ_T_* use the shock wave physics experimental technique by using a flat plate impact test with a lightweight air cannon. The pressure range covered by the chemical explosion contact blast is crossed with the flat plate impact test, so the required parameters are referred to the flat plate impact test [37]. Therefore, the above formula takes parameters as shown in Table 12.

**Figure 15:**
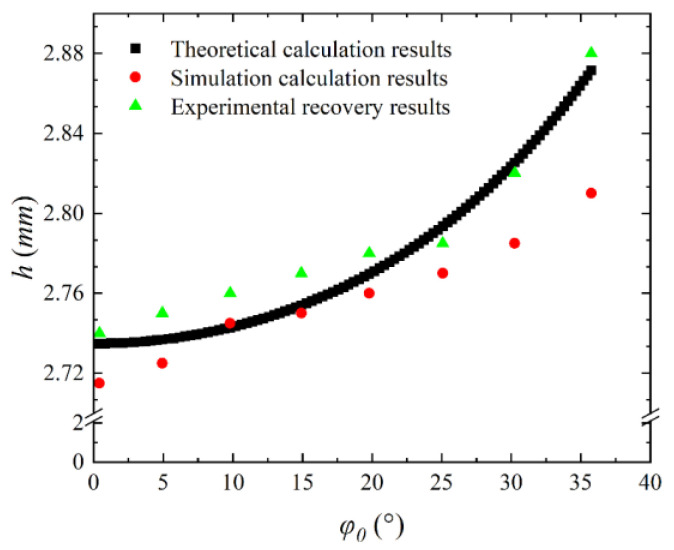
Variation of fracture length with incident angle.

The value of *C* is 4520 m/s by Equation (17). In the case of neglecting the attenuation of the shock wave in the rupture, the variation curve of the fracture length (*h*) from the rupture far-exploding surface (red) with the angle of incidence is shown in Figure 15 according to Equation (15) and the calculation results of the pressure amplitude in the previous section.

The results of the simulation and the experiments are represented by the green and orange ranges, as shown in Figure 15. The theoretical results of *h* (2.73–2.86 mm) are consistent with the results of experimental recovery measurements (2.74–2.88 mm) and the results of simulated (2.71–2.78 mm), which confirms the reliability of the analysis. According to the recovered fracture samples, it is found that the fracture surface is not a relatively smooth surface in the height direction, and its fracture surface is uneven. This is because the fracture of the material is a process of damage accumulation, which can be expressed by the combination of macro–micro simulation [38,39].

Therefore, it is difficult to accurately calculate the position of different fractures for materials during the accumulation process. Then, the approximate theoretical calculation is used here, and the measurement method takes the average height of the recovered fracture samples. Thus, the results of the theoretical calculations can account for a certain degree of agreement with the simulation and test results.

## 5. Summary

The effect of surface electroplating on the fragment deformation behavior under contact explosion was analyzed by the combination of theory, experiment, and simulation in this study. The following conclusions can be drawn:

1. The coating prevents the molten state and plastic flow of the fragments in the radial direction. In contrast to Kevlar/epoxy lining, the coating not only prevents the molten state of fragments due to collisions between neighboring fragments but also prevents the plastic flow of the fragments by preventing their contact with high-temperature gas;

2. The coating changes the deformation behavior. Because the wave impedance of the nickel coating is greater than that of the fragment, it changes from the stretch zone of the original interface with the air to the compression zone at the interface between the fragment and the coating, which solves the problem of tensile fracture of the fragments on the far-exploding surface (free surface);

3. By simplifying the shock wave as one-dimensional stress propagation, the axial fracture length of the uncoated samples is between 2.73–2.86 mm. The error is less than 6% compared with the recovered samples from the test method of UC-10L. This can prove the applicability of the theory considering the error caused by the cumulative fracture of the material microdamage.

## Figures and Tables

**Table 1:** Coating solution formulations (g/L).

Type	NiSO_4_	NiCl_2_	NaCl_2_	H_3_BO_3_	C_7_H_5_NO_3_S	C_12_H_25_SO_4_Na	C_6_H_5_SO_2_Na	C_4_H_6_O_2_
Coating solution A	300	40	/	40	0.8	0.05	/	/
Coating solution B	300	/	10	35	0.8	0.05	0.5	0.4

**Table 2:** Test arrangement.

Test Method	Fragmentation Type	Lining Thickness
UC-10L	uncoated samples	10 mm
C-10L	coated samples	10 mm

**Table 3:** Material parameters of nylon.

*ρ/*g∙cm^−3^	*E/*GPa	*v*	*σ*_0_/MPa	*E_tan_/*MPa	*F_s_*
1.1	4.5	0.375	98	4.5	1.0

**Table 4:** Material parameters of explosive.

*ρ/*g∙cm^−3^	*D*/m∙s^−1^	*P_CJ_/*GPa	*a*/m∙s^−2^	*b*	*R*_1_	*R*_2_	*OMEG*
1.68	8800	29.75	4818	0.213	4.602	1.653	0.5

**Table 5:** Material parameters of air.

*ρ/*g∙cm^−3^	*C*_0_/GPa	*C*_1_/GPa	*C*_2_/GPa	*C*_3_	*C*_4_	*C*_5_	*C*_6_
1.1845	0	0	0	0	0.4	0.4	0

**Table 6:** Material parameters of lining.

*ρ/*g∙cm^−3^	*E*_1_/GPa	*E*_2_/GPa	*E*_3_/GPa	*V*_12_	*V*_13_	*V*_23_
1.44	18.5	18.5	6	0.25	0.33	0.33

**Table 7:** Material parameters of the zirconium fragment.

*ρ/*g∙cm^−3^	*C_v_/*J∙kg^−1^∙K^−1^	*T_emit_/*K	*A/*MPa	*B/*MPa	*n*	*C*	*m*
6.484	270	1473	303.8	549.12	0.65	0.027	0.827

**Table 8:** Material parameters of the Ni coating.

*ρ/*g∙cm^−3^	*C_v_/*J∙kg^−1^∙K^−1^	*T_emit_/*K	*A/*MPa	*B/*MPa	*n*	*C*	*m*
8.9	446	1726	163	648	0.33	0.006	1.44

**Table 9:** Explosive composition and its mass fraction.

*μ* (RDX)%	*μ* (Nitrotoluene)%	*μ* (Vinyl Acetate) %	Stearic Acid
95	3	2	trace

**Table 10:** The material parameters used in calculations.

*ρ*_0_/g∙cm^−3^	*ρ*_m0_/g∙cm^−3^	*a*/m∙s^−2^	*b*	*D*/m∙s^−2^	*k*_0_/RDX	*k*_0_/Vinyl Acetate	*k*_0_/Nitrotoluene
1.68	1.273	2610	1.42	8800	2.65	2.78	2.78

Note: *ρ*_0_ is the density of the explosive; *ρ_m*0*_* is the density of Kevlar/epoxy composite lining; *a* and *b* are the empirical constants of the lining’s shock compression relation; *D* is the detonation wave velocity; *k*_0_ is a part of *k*, which is related to density [31,32].

**Table 11:** Parameters for different mediums.

Medium Type	*ρ_m*0*_/*g∙cm^−3^	*a/*m∙s^−2^	*b*
Nickel coating	8.9	4590	1.44
Zirconium fragment	6.5	4240	1.015

**Table 12:** Table of parameters used for calculation.

*ρ/*g∙cm^−3^	*E*/GPa	*υ*	*σ_T_*/GPa	*d/*mm
6.5	101	0.31	1.69	72

## Data Availability

Not applicable.
